# Nitro­syltris(pyridine-2-thiol­ato-κ^2^ *N*,*S*)molybdenum(II) dihydrate

**DOI:** 10.1107/S1600536809043712

**Published:** 2009-10-28

**Authors:** Toshiaki Yonemura

**Affiliations:** a Department of Applied Science, Faculty of Science, Kochi University, Akebono-cho, Kochi 780-8520, Japan

## Abstract

In the title compound, [Mo(C_5_H_4_NS)_3_(NO)]·2H_2_O, the Mo atom is coordinated by a nitrosyl ligand and three monoanionic *N*,*S*-bidentate ligands in a distorted MoN_4_S_3_ penta­gonal-bipyramidal mol­ecular geometry. The pyridine N atom of one pyridine-2-thiol­ate (pyt) ligand is coordinated to the Mo atom in the *trans* position relative to the NO ligand [N(pyt)—Mo—N(NO) = 170.62 (19)°]. The compound has *C*_s_ symmetry, with a mirror plane that includes one pyt ring and the NO group. The S—Mo—N(NO) and N(pyt)—Mo—N(NO) angles [97.24 (12) and 91.87 (8)°, respectively] are large relative to the ideal angles of 90°. In the crystal, the mol­ecules pack in a zigzag arrangement. The cavities between the mol­ecules are occupied by disordered water mol­ecules of crystallization.

## Related literature

For the synthesis and chemistry of similar nitrosyl, pyridine­thilato, or pyrimidine­thiol­ato derivative complexes, see: Halpenny & Mascharak (2009 ▶); Rose *et al.* (2007 ▶); Cini *et al.* (2003 ▶); Maurya *et al.* (2006 ▶); Kunkely & Vogler (2003 ▶); Ford *et al.* (1998 ▶); Proust *et al.* (1994 ▶); Ardon & Cohen (1993 ▶); Calderon *et al.* (1969 ▶); Yonemura *et al.* (2006 ▶, 2001 ▶); Bucher *et al.* (2008 ▶).
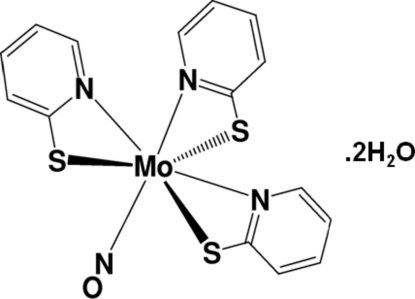


## Experimental

### 

#### Crystal data

[Mo(C_5_H_4_NS)_3_(NO)]·2H_2_O
*M* *_r_* = 492.44
Orthorhombic, 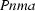

*a* = 15.7519 (16) Å
*b* = 14.8889 (14) Å
*c* = 9.0535 (12) Å
*V* = 2123.3 (4) Å^3^
*Z* = 4
Mo *K*α radiation
μ = 0.93 mm^−1^
*T* = 296 K
0.45 × 0.40 × 0.25 mm

#### Data collection

Rigaku AFC-7S diffractometer
Absorption correction: ψ scan (North *et al.*, 1968 ▶) *T*_min_ = 0.727, *T*_max_ = 0.792
3681 measured reflections
2540 independent reflections
2088 reflections with *I* > 2σ(*I*)
*R*_int_ = 0.023
3 standard reflections every 150 reflections intensity decay: 1.3%

#### Refinement

*R*[*F* ^2^ > 2σ(*F* ^2^)] = 0.039
*wR*(*F* ^2^) = 0.126
*S* = 1.13
2540 reflections
144 parameters
H-atom parameters constrained
Δρ_max_ = 1.14 e Å^−3^
Δρ_min_ = −0.64 e Å^−3^

### 

Data collection: *WinAFC* (Rigaku/MSC, 2000 ▶); cell refinement: *WinAFC*; data reduction: *CrystalStructure* (Rigaku/MSC, 2007 ▶); program(s) used to solve structure: *SHELXS97* (Sheldrick, 2008 ▶); program(s) used to refine structure: *SHELXL97* (Sheldrick, 2008 ▶); molecular graphics: *ORTEP-3 for Windows* (Farrugia, 1997 ▶); software used to prepare material for publication: *CrystalStructure*.

## Supplementary Material

Crystal structure: contains datablocks global, I. DOI: 10.1107/S1600536809043712/su2152sup1.cif

Structure factors: contains datablocks I. DOI: 10.1107/S1600536809043712/su2152Isup2.hkl

Additional supplementary materials:  crystallographic information; 3D view; checkCIF report

## Figures and Tables

**Table:** 

Mo1—S1	2.5240 (12)
Mo1—S2	2.4815 (16)
Mo1—N1	2.218 (5)
Mo1—N2	2.228 (3)
Mo1—N3	1.777 (5)

**Table:** 

S1—Mo1—S2	138.29 (3)
S1—Mo1—N1	90.40 (10)
S1—Mo1—N3	97.24 (12)
S2—Mo1—N2	80.58 (7)
S1^i^—Mo1—S2	138.29 (3)
N1—Mo1—N2	86.66 (8)
N1—Mo1—N3	170.62 (19)
N2—Mo1—N3	91.87 (8)

Symmetry code: (i) 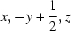.

**Fig. 1.**
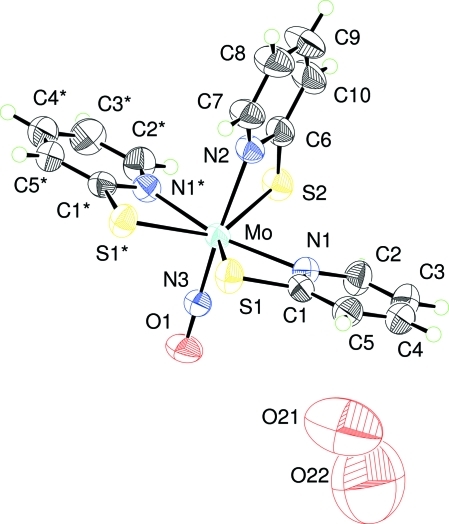
A view of the molecular structure of the title compound, showing the atom-labelling scheme and 50% probability displacement ellipsoids. Atoms related by the mirror symmetry are marked by *; symmetry operation: x, -y+1/2, z.

**Fig. 2.**
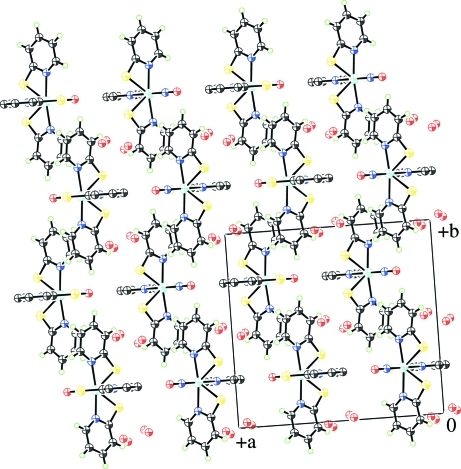
Crystal packing diagram of the title compound viewed along the c axis (some H-atoms have been omitted for clarity).

## supplementary crystallographic information

### Comment

In recent years, pyridinethiolate- or pyrimidinethiolate-type ligands and their complexes have been investigated as antimetabolite and antiviral agents, as well as for their unique photochemical properties (Halpenny & Mascharak, 2009; Rose *et al.*, 2007; Cini *et al.*, 2003). For example, attempts to regulate NO *in vivo* have prompted studies of NO scavengers and NO-releasing drugs. Although some photoinduced NO-releasing reactions of mononitrosyl complexes have been reported, relatively little is known about the analogous reactions of dinitrosyl complexes in this respect (Maurya *et al.*, 2006; Kunkely & Vogler, 2003; Ford *et al.*, 1998). We previously reported on the preparation, characterization and interesting photo-dimerization reactions of some dinitrosyl-molybdenum complexes containing thiolate ligands, which were accompanied by NO cleavage (Yonemura *et al.*, 2001, 2006). This highlighted the need to further study the reactivities and properties of these dinitrosyl-molybdenum complexes. That communication described a novel reaction of dinitrosyl-molybdenum [Mo(bidentate-*N,S*)_2_(NO)_2_]-type complexes with PPh_3_ (Yonemura, *et al.*, 2006). This reaction, which uses pyridine-2-thiolate (pyt) as a thiolate ligand, was shown to form [Mo(pyt)_3_(NO)], [{(ON)Mo(pyt)_2_}_2_(µ-OH)_2_], and Ph_3_PO. In this paper, we report on the structure of [Mo(pyt)_3_(NO)] Dihydrate.

In the title compound the molybdenum atom is coordinated to a nitrosyl ligand and three monoanionic *N,S*- bidentate ligands, producing a distorted MoN_5_S_2_ pentagonal bipyramidal molecular geometry (Fig. 1 and Table 1). The geometrical parameters are available in the archived CIF. This complex is derived from the elimination of one NO ligand from [Mo(pyt)_2_(NO)_2_] and the introduction of a third pyt ligand, giving rise to a Mo atom surrounded by three pyt ligands and one NO ligand. The complex adopts a seven-coordinate structure with a distorted pentagonal bipyramidal coordination geometry about the Mo atom. Both the N and S atoms of two pyt ligands and an S atom of the third pyt ligand occupy the equatorial positions of the complex. The remaining N-atom of the third pyt ligand occupies one of the axial sites. The NO ligand occupies the other axial site in its linear mode [Mo1—N3—O1 = 179.6 (4)°], indicating that the NO ligand is coordinated as NO^+^ (Proust *et al.*, 1994; Ardon & Cohen, 1993; Calderon *et al.*,  1969). Therefore, the oxidation state of the molybdenum atom in the title compound is formally +II; that is, the molybdenum atom is oxidized from 0 to +II.

The Mo—S distances are 2.5240 (12) and 2.4815 (16) Å, compared to 2.497 (3) and 2.477 (3) Å in complex [Mo(pymt)_2_(NO)_2_] (Yonemura *et al.*, 2001), and 2.4870 (7) Å in [Mo(aet)_2_(NO)_2_] (Bucher *et al.*, 2008). In this latter complex, the Mo—N2 distance ( 2.228 (3) Å), corresponding to the N *trans* to the NO ligand, is almost the same as the other Mo—N distance in the title compound (Mo1—N1 = 2.218 (5) Å). The Mo1—NO distance ( 1.777 (5) Å) in the title compound is significantly shorter than those in complex es [Mo(pymt)_2_(NO)_2_] (1.814 (8) and 1.84 (1) Å), and [Mo(aet)_2_(NO)_2_] (1.828 (2) and 1.837 (2) Å). However, the Mo1—NO distance is almost the same as that in complex [{(ON)Mo(pyt)_2_}_2_(µ-OH)_2_], that is 1.756 (2) Å (Yonemura *et al.*, 2001). The S1—Mo1—N3(NO) and N1—Mo1—N3(NO) angles (97.24 (12) and 91.87 (8)°, respectively) are large compared to the corresponding angles (95.71 (6), 94.48 (7) and 86.36 (8), 88.12 (8)°, respectively) in [{(ON)Mo(pyt)_2_}_2_(µ-OH)_2_].

In the crystal the molecules pack in a zigzag arrangement (Fig. 2). The cavities between the molecules are occupied by disordered water molecules of crystallization.

### Experimental

A solution of [Mo(pyt)_2_(NO)_2_] (0.25 g, 0.65 mmol) in *N*,*N*-dimethylformamide (DMF) and PPh_3_ (0.37 g, 1.41 mmol) in tetrahydrofuran (THF) was stirred at rt for 4 days to produce an orange solution. Yellow precipitates of [{(ON)Mo(pyt)_2_}_2_(µ-OH)_2_] and [Mo(pyt)_3_(NO)] were obtained by allowing the reaction solution to stand in a refrigerator for a few days. The resulting orange-yellow crystals were collected by filtration. The filtrate was then poured into water, and the precipitate produced was collected by filtration and recrystallized from an acetone solution to give orange-yellow crystals of the title compound (23% yield). Anal. Calcd for [Mo(pyt)_3_(NO)] = C_16_H_16_MoN_4_O_2_S_3_: C 25.53, H 1.55, N 22.13%, Found: C 25.70, H 1.61, N 21.98%. IR [KBr; ν_max_,, cm^-1^]: 1644 (NO), 1582, 1551(CC, CN), 1447, 1420 (NC, CH), 1260 (CS). ^13^C NMR (acetone-d_6_): *δ* 176.5, 149.1, 148.2, 140.3, 140.1, 126.8, 126.5, 119.9, 118.7.

### Refinement

The water molecules of solvent of crystallization are disordered with occupancies of 0.5 each, and it was not possible to locate their H-atoms. The C-bound H-atom were included in calculated positions and treated as riding: C—H = 0.93 Å, with *U*_iso_(H) = 1.2*U*_eq_(C).

### Crystal data

**Table:**

[Mo(C_5_H_4_NS)_3_(NO)]·2H_2_O	*F*(000) = 992.00
*M**_r_* = 492.44	*D*_x_ = 1.540 Mg m^−^^3^
Orthorhombic, *P**n**m**a*	Mo *K*α radiation, λ = 0.71069 Å
Hall symbol: -P 2ac 2n	Cell parameters from 25 reflections
*a* = 15.7519 (16) Å	θ = 15.4–17.4°
*b* = 14.8889 (14) Å	µ = 0.93 mm^−^^1^
*c* = 9.0535 (12) Å	*T* = 296 K
*V* = 2123.3 (4) Å^3^	Prismatic, orange
*Z* = 4	0.45 × 0.40 × 0.25 mm

### Data collection

**Table:**

Rigaku AFC-7S diffractometer	*R*_int_ = 0.023
ω–2θ scans	θ_max_ = 27.5°
Absorption correction: ψ scan (North *et al.*, 1968)	*h* = 0→20
*T*_min_ = 0.727, *T*_max_ = 0.792	*k* = −10→19
3681 measured reflections	*l* = −11→6
2540 independent reflections	3 standard reflections every 150 reflections
2088 reflections with *F*^2^ > 2σ(*F*^2^)	intensity decay: −1.3%

### Refinement

**Table:**

Refinement on *F*^2^	H-atom parameters constrained
*R*[*F*^2^ > 2σ(*F*^2^)] = 0.039	*w* = 1/[σ^2^(*F*_o_^2^) + (0.0621*P*)^2^ + 2.5132*P*] where *P* = (*F*_o_^2^ + 2*F*_c_^2^)/3
*wR*(*F*^2^) = 0.126	(Δ/σ)_max_ = 0.002
*S* = 1.13	Δρ_max_ = 1.14 e Å^−^^3^
2540 reflections	Δρ_min_ = −0.64 e Å^−^^3^
144 parameters	Extinction correction: *SHELXL97* (Sheldrick, 2008)
0 restraints	Extinction coefficient: 0.0029 (5)

### Special details

**Table:**

Geometry. Bond distances, angles etc. have been calculated using the rounded fractional coordinates. All su's are estimated from the variances of the (full) variance-covariance matrix. The cell esds are taken into account in the estimation of distances, angles and torsion angles
Refinement. Refinement was performed using all reflections. The weighted *R*-factor (*wR*) and goodness of fit (*S*) are based on *F*^2^. *R*-factor (gt) are based on *F*. The threshold expression of *F*^2^ > 2.0 σ(*F*^2^) is used only for calculating *R*-factor (gt).

### Fractional atomic coordinates and isotropic or equivalent isotropic displacement parameters (Å^2^)

**Table:**

	*x*	*y*	*z*	*U*_iso_*/*U*_eq_	Occ. (<1)
Mo1	0.17875 (3)	0.25000	0.95698 (4)	0.0359 (1)	
S1	0.07755 (7)	0.15176 (7)	1.10056 (12)	0.0512 (3)	
S2	0.25067 (10)	0.25000	0.71312 (17)	0.0516 (4)	
O1	0.3191 (3)	0.25000	1.1749 (5)	0.0667 (14)	
N1	0.0907 (3)	0.25000	0.7658 (5)	0.0427 (12)	
N2	0.1905 (2)	0.1024 (2)	0.9222 (3)	0.0427 (9)	
N3	0.2627 (3)	0.25000	1.0881 (5)	0.0423 (12)	
C1	0.1292 (2)	0.0662 (2)	1.0080 (4)	0.0420 (10)	
C2	0.1159 (3)	−0.0257 (2)	1.0145 (4)	0.0520 (12)	
C3	0.1680 (3)	−0.0801 (3)	0.9319 (5)	0.0627 (14)	
C4	0.2315 (3)	−0.0437 (3)	0.8467 (5)	0.0653 (16)	
C5	0.2415 (2)	0.0478 (3)	0.8434 (5)	0.0560 (12)	
C6	0.1452 (4)	0.25000	0.6512 (6)	0.0477 (14)	
C7	0.0065 (4)	0.25000	0.7415 (8)	0.0570 (17)	
C8	−0.0257 (5)	0.25000	0.5987 (9)	0.077 (3)	
C9	0.0308 (6)	0.25000	0.4820 (8)	0.080 (3)	
C10	0.1160 (5)	0.25000	0.5066 (7)	0.066 (2)	
O21	−0.0333 (11)	0.4696 (10)	0.609 (2)	0.258 (12)	0.500
O22	0.0678 (7)	0.5119 (9)	0.5891 (12)	0.128 (5)	0.500
H1	0.07300	−0.04980	1.07300	0.0630*	
H2	0.16030	−0.14210	0.93360	0.0750*	
H3	0.26730	−0.08070	0.79200	0.0780*	
H4	0.28430	0.07260	0.78550	0.0670*	
H5	−0.03070	0.25000	0.82130	0.0690*	
H6	−0.08390	0.25000	0.58200	0.0920*	
H7	0.01040	0.25000	0.38570	0.0960*	
H8	0.15400	0.25000	0.42800	0.0790*	
H9	−0.01120	0.48460	0.54280	0.3170*	0.500
H10	−0.05690	0.48240	0.65730	0.3170*	0.500
H11	0.02840	0.51090	0.60270	0.1900*	0.500
H12	0.08240	0.49480	0.49830	0.1900*	0.500

### Atomic displacement parameters (Å^2^)

**Table:**

	*U*^11^	*U*^22^	*U*^33^	*U*^12^	*U*^13^	*U*^23^
Mo1	0.0365 (2)	0.0382 (2)	0.0330 (2)	0.0000	0.0017 (2)	0.0000
S1	0.0579 (5)	0.0446 (5)	0.0510 (5)	−0.0049 (4)	0.0178 (4)	−0.0019 (4)
S2	0.0495 (7)	0.0588 (8)	0.0464 (7)	0.0000	0.0145 (6)	0.0000
O1	0.066 (2)	0.074 (3)	0.060 (2)	0.0000	−0.025 (2)	0.0000
N1	0.045 (2)	0.049 (2)	0.034 (2)	0.0000	−0.0012 (18)	0.0000
N2	0.0440 (16)	0.0440 (17)	0.0401 (15)	0.0014 (13)	0.0003 (12)	−0.0030 (13)
N3	0.044 (2)	0.042 (2)	0.041 (2)	0.0000	−0.0001 (19)	0.0000
C1	0.0457 (19)	0.0416 (18)	0.0387 (17)	0.0002 (15)	−0.0037 (15)	−0.0016 (14)
C2	0.063 (2)	0.044 (2)	0.049 (2)	−0.0051 (19)	−0.0008 (19)	0.0026 (17)
C3	0.085 (3)	0.039 (2)	0.064 (2)	0.004 (2)	−0.011 (2)	−0.0020 (19)
C4	0.072 (3)	0.051 (2)	0.073 (3)	0.017 (2)	0.007 (2)	−0.008 (2)
C5	0.054 (2)	0.056 (2)	0.058 (2)	0.0076 (19)	0.0063 (19)	−0.004 (2)
C6	0.057 (3)	0.047 (2)	0.039 (2)	0.0000	0.005 (2)	0.0000
C7	0.047 (3)	0.068 (3)	0.056 (3)	0.0000	−0.003 (2)	0.0000
C8	0.070 (4)	0.099 (6)	0.061 (4)	0.0000	−0.023 (3)	0.0000
C9	0.095 (6)	0.103 (6)	0.043 (3)	0.0000	−0.019 (3)	0.0000
C10	0.084 (5)	0.079 (4)	0.034 (2)	0.0000	0.006 (3)	0.0000
O21	0.213 (18)	0.132 (13)	0.43 (3)	0.100 (12)	−0.21 (2)	−0.171 (17)
O22	0.104 (7)	0.164 (11)	0.117 (7)	0.009 (7)	−0.024 (6)	0.001 (8)

### Geometric parameters (Å, °)

**Table:**

Mo1—S1	2.5240 (12)	N2—C5	1.347 (5)
Mo1—S2	2.4815 (16)	C1—C2	1.386 (4)
Mo1—N1	2.218 (5)	C2—C3	1.374 (6)
Mo1—N2	2.228 (3)	C3—C4	1.375 (7)
Mo1—N3	1.777 (5)	C4—C5	1.372 (6)
Mo1—S1^i^	2.5240 (12)	C6—C10	1.388 (9)
Mo1—N2^i^	2.228 (3)	C7—C8	1.389 (11)
S1—C1	1.728 (3)	C8—C9	1.381 (12)
S2—C6	1.753 (6)	C9—C10	1.360 (12)
O1—N3	1.186 (7)	C2—H1	0.9300
O21—O22	1.72 (2)	C3—H2	0.9300
O21—H10	0.6000	C4—H3	0.9300
O21—H9	0.7300	C5—H4	0.9300
O22—H12	0.8900	C7—H5	0.9300
O22—H11	0.6300	C8—H6	0.9300
N1—C6	1.347 (7)	C9—H7	0.9300
N1—C7	1.344 (8)	C10—H8	0.9300
N2—C1	1.351 (4)		
			
S1—Mo1—S2	138.29 (3)	Mo1—N3—O1	179.6 (4)
S1—Mo1—N1	90.40 (10)	N2—C1—C2	121.8 (3)
S1—Mo1—N2	63.49 (8)	S1—C1—N2	108.7 (2)
S1—Mo1—N3	97.24 (12)	S1—C1—C2	129.5 (3)
S1—Mo1—S1^i^	70.83 (4)	C1—C2—C3	118.0 (4)
S1—Mo1—N2^i^	134.18 (8)	C2—C3—C4	120.5 (4)
S2—Mo1—N1	65.87 (13)	C3—C4—C5	119.2 (4)
S2—Mo1—N2	80.58 (7)	N2—C5—C4	121.3 (4)
S2—Mo1—N3	104.75 (15)	N1—C6—C10	121.0 (6)
S1^i^—Mo1—S2	138.29 (3)	S2—C6—N1	111.0 (4)
S2—Mo1—N2^i^	80.58 (7)	S2—C6—C10	128.0 (5)
N1—Mo1—N2	86.66 (8)	N1—C7—C8	120.8 (6)
N1—Mo1—N3	170.62 (19)	C7—C8—C9	118.5 (7)
S1^i^—Mo1—N1	90.40 (10)	C8—C9—C10	120.7 (7)
N1—Mo1—N2^i^	86.66 (8)	C6—C10—C9	118.8 (6)
N2—Mo1—N3	91.87 (8)	C1—C2—H1	121.00
S1^i^—Mo1—N2	134.18 (8)	C3—C2—H1	121.00
N2—Mo1—N2^i^	161.13 (11)	C2—C3—H2	120.00
S1^i^—Mo1—N3	97.24 (12)	C4—C3—H2	120.00
N2^i^—Mo1—N3	91.87 (8)	C5—C4—H3	120.00
S1^i^—Mo1—N2^i^	63.49 (8)	C3—C4—H3	120.00
Mo1—S1—C1	83.12 (11)	N2—C5—H4	119.00
Mo1—S2—C6	81.48 (19)	C4—C5—H4	119.00
H9—O21—H10	142.00	N1—C7—H5	120.00
H11—O22—H12	115.00	C8—C7—H5	120.00
Mo1—N1—C6	101.7 (4)	C7—C8—H6	121.00
C6—N1—C7	120.2 (5)	C9—C8—H6	121.00
Mo1—N1—C7	138.1 (4)	C8—C9—H7	120.00
Mo1—N2—C5	136.0 (3)	C10—C9—H7	120.00
C1—N2—C5	119.3 (3)	C9—C10—H8	121.00
Mo1—N2—C1	104.6 (2)	C6—C10—H8	121.00
			
S2—Mo1—S1—C1	32.54 (14)	Mo1—S1—C1—N2	1.4 (2)
N1—Mo1—S1—C1	85.19 (14)	Mo1—S1—C1—C2	−179.6 (4)
N2—Mo1—S1—C1	−0.87 (14)	Mo1—S2—C6—N1	0.00
N3—Mo1—S1—C1	−89.34 (15)	Mo1—S2—C6—C10	180.00
S1^i^—Mo1—S1—C1	175.48 (12)	Mo1—N1—C6—S2	0.00
N2^i^—Mo1—S1—C1	170.92 (16)	Mo1—N1—C6—C10	180.00
S1—Mo1—S2—C6	60.58 (6)	C7—N1—C6—S2	180.00
N1—Mo1—S2—C6	0.00	C7—N1—C6—C10	0.00
N2—Mo1—S2—C6	90.55 (8)	Mo1—N1—C7—C8	180.00
N3—Mo1—S2—C6	−180.00	C6—N1—C7—C8	0.00
S1—Mo1—N1—C6	−144.58 (2)	Mo1—N2—C1—S1	−1.6 (3)
S1—Mo1—N1—C7	35.42 (2)	Mo1—N2—C1—C2	179.3 (3)
S2—Mo1—N1—C6	0.00	C5—N2—C1—S1	177.5 (3)
S2—Mo1—N1—C7	180.00	C5—N2—C1—C2	−1.6 (5)
N2—Mo1—N1—C6	−81.17 (8)	Mo1—N2—C5—C4	179.9 (3)
N2—Mo1—N1—C7	98.83 (8)	C1—N2—C5—C4	1.1 (6)
S1—Mo1—N2—C1	1.14 (19)	S1—C1—C2—C3	−178.1 (3)
S1—Mo1—N2—C5	−177.8 (4)	N2—C1—C2—C3	0.8 (6)
S2—Mo1—N2—C1	−157.1 (2)	C1—C2—C3—C4	0.4 (6)
S2—Mo1—N2—C5	24.1 (3)	C2—C3—C4—C5	−0.9 (7)
N1—Mo1—N2—C1	−91.0 (2)	C3—C4—C5—N2	0.2 (7)
N1—Mo1—N2—C5	90.1 (4)	S2—C6—C10—C9	180.00
N3—Mo1—N2—C1	98.3 (3)	N1—C6—C10—C9	0.00
N3—Mo1—N2—C5	−80.6 (4)	N1—C7—C8—C9	0.00
S1^i^—Mo1—N2—C1	−3.7 (3)	C7—C8—C9—C10	0.00
S1^i^—Mo1—N2—C5	177.4 (3)	C8—C9—C10—C6	0.00

Symmetry codes: (i) *x*, −*y*+1/2, *z*.

### Hydrogen-bond geometry (Å, °)

**Table:**

*D*—H···*A*	*D*—H	H···*A*	*D*···*A*	*D*—H···*A*
O21—H9···O22	0.73	1.37	1.72 (2)	106
C5—H4···S2	0.93	2.77	3.237 (5)	112
